# Text mining-based identification of promising miRNA biomarkers for diabetes mellitus

**DOI:** 10.3389/fendo.2023.1195145

**Published:** 2023-07-25

**Authors:** Xin Li, Andrea Dai, Richard Tran, Jie Wang

**Affiliations:** ^1^Central Hospital Affiliated to Shandong First Medical University, Ophthalmology Department, Jinan, Shandong, China; ^2^Oakland University William Beaumont School of Medicine, Rochester, MI, United States; ^3^University of Chicago, Master’s Program in Computer Science, Chicago, IL, United States; ^4^Syracuse University, Applied Data Science Program, Syracuse, NY, United States; ^5^MDSight, LLC, Brookeville, MD, United States

**Keywords:** microRNA, text mining, machine learning, diabetes, miR-146

## Abstract

**Introduction:**

MicroRNAs (miRNAs) are small, non-coding RNAs that play a critical role in diabetes development. While individual studies investigating the mechanisms of miRNA in diabetes provide valuable insights, their narrow focus limits their ability to provide a comprehensive understanding of miRNAs’ role in diabetes pathogenesis and complications.

**Methods:**

To reduce potential bias from individual studies, we employed a text mining-based approach to identify the role of miRNAs in diabetes and their potential as biomarker candidates. Abstracts of publications were tokenized, and biomedical terms were extracted for topic modeling. Four machine learning algorithms, including Naïve Bayes, Decision Tree, Random Forest, and Support Vector Machines (SVM), were employed for diabetes classification. Feature importance was assessed to construct miRNA-diabetes networks.

**Results:**

Our analysis identified 13 distinct topics of miRNA studies in the context of diabetes, and miRNAs exhibited a topic-specific pattern. SVM achieved a promising prediction for diabetes with an accuracy score greater than 60%. Notably, miR-146 emerged as one of the critical biomarkers for diabetes prediction, targeting multiple genes and signal pathways implicated in diabetic inflammation and neuropathy.

**Conclusion:**

This comprehensive approach yields generalizable insights into the network miRNAs-diabetes network and supports miRNAs’ potential as a biomarker for diabetes.

## Introduction

Diabetes is a prevalent endocrine disease characterized by elevated blood glucose levels, which has rapidly grown in incidence and has become a global health concern (1). The long-term consequences of diabetes include both microvascular and macrovascular complications, which pose a severe threat to the health and well-being of individuals. Among diabetic patients, microvascular complications such as diabetic kidney disease, diabetic retinopathy, diabetic neuropathy, and diabetic foot are prevalent, while macrovascular complications such as cardiovascular disease can lead to fatal outcomes (2–4). The substantial impact of diabetes on morbidity, mortality, and quality of life places a significant burden on healthcare systems worldwide.

microRNAs (miRNAs) are a class of small, non-coding RNAs that mediate post transcriptional gene silencing (5). These regulatory molecules have emerged as crucial players in orchestrating cellular responses to physiological perturbations and disease conditions (6, 7). Recent research has highlighted the pivotal role of miRNAs in the pathogenesis of diabetes and its associated complications (8). For instance, miRNAs have demonstrated indispensable roles in pancreatic beta cells, regulating their response to metabolic, genetic, and inflammatory stressors (9–12). This underscores the importance of miRNAs in diabetes management and emphasizes their potential as therapeutic targets for the treatment of diabetes.

PubMed is a widely accessible research interface housing an impressive repository of approximately 35 million medical publications as of 2021, including a substantial number of studies investigating various aspects of diabetes. While individual studies investigating the mechanisms of miRNA in diabetes provide valuable insights, their narrow focus limits their ability to provide a comprehensive understanding of miRNAs’ role in diabetes pathogenesis and complications. To achieve a more complete understanding of the complex interactions between miRNAs and diabetes, it is essential to adopt a comprehensive approach that considers the diverse range of factors involved. By utilizing text mining techniques to identify miRNA and diabetes-related literature, we can collect information from multiple studies and establish a more holistic perspective. This approach enables researchers to identify patterns and gaps in the literature and generate new hypotheses to guide future investigations. Ultimately, applying this comprehensive approach contributes to a more profound and thorough understanding of the role of miRNAs in diabetes and its associated complications.

## Method

**Figure 1:**
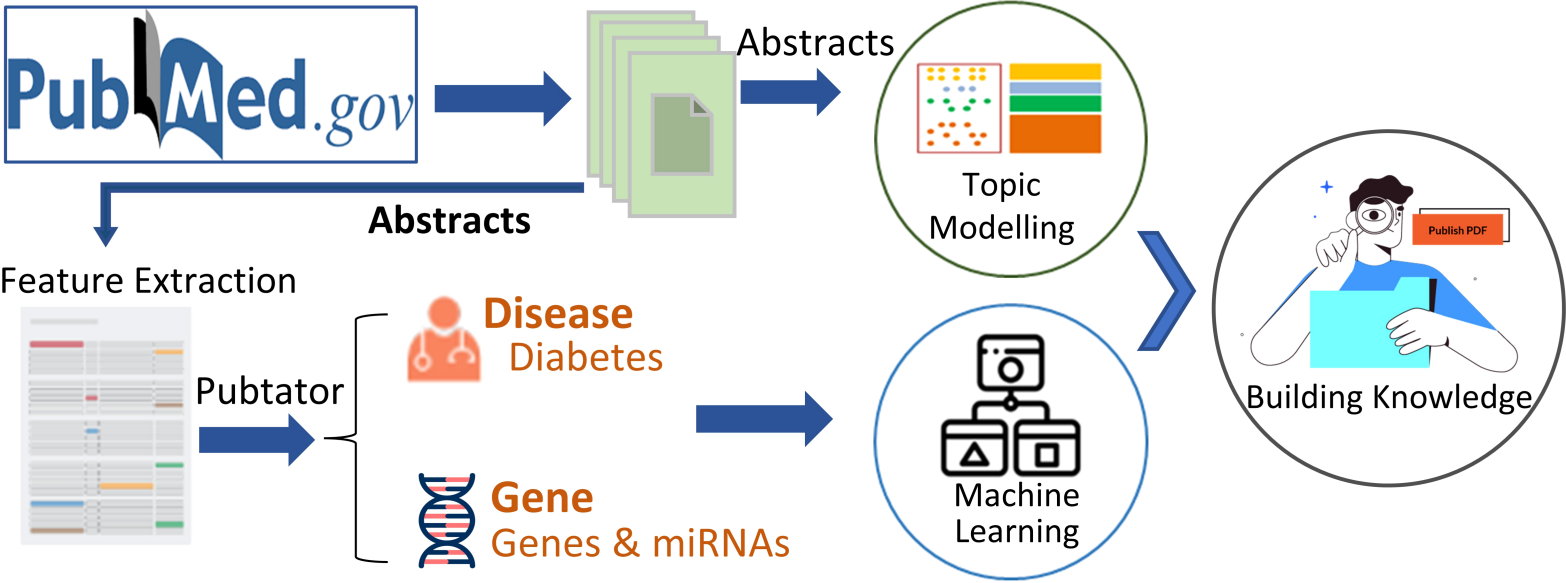
Study Design and workflow.

The strategy was shown in **Figure 1**.

### PubMed corpus

PubMed was utilized to retrieve literature information for studies investigating miRNAs in the context of diabetes, spanning the period from January 1, 1993, to March 5, 2023. To assess the specificity and robustness of our machine learning model in distinguishing between diabetes-related and non-diabetes-related studies, miRNA study in diabetes dataset and miRNA study in non-diabetes dataset (referred to as the negative control) were prepared. The search criterion employed for the diabetes dataset was “miRNA” [Title/Abstract] AND “Diabetes” [Title/Abstract], while for the negative control dataset, it was “miRNA” [Title/Abstract] NOT “Diabetes” [Title/Abstract]. The following data were collected for this study: PMID, Publication Date, Publication Type, First Author, Journal Name, Literature Title, and Literature Abstract. Specifically, the study focused on specific publication types including “Case Reports”, “Clinical Study”, “Clinical Trail”, “Comparative Study”, “English Abstract”, “Evaluation Study”, “Journal Article”, “Letter”, and “Preprint”, while excluded “Retraction of Publication”, “Published Erratum”, “Editorial”, “News”, “Dataset”, “Clinical trial protocol”, “Review”, and “Systematic review”.

### Topic modeling

Topic modeling is a statistical technique used in natural language processing to uncover hidden patterns and structures within a large corpus. The algorithm employed in the current study for topic modeling is Latent Dirichlet Allocation (LDA), which assumes that each document in the corpus is a mixture of multiple topics, and each topic is characterized by a distribution of words from the abstracts.

### Biomedical term tagging

In this text mining study, the focus was placed on literature titles and abstracts due to the crucial information they contain and their widespread availability. To tag biomedical terms (including miRNAs) within the literature, two methods were employed: PubTator and Regex.

PubTator: PubTator is a web-based biomedical named entity recognition (NER) system (13) specifically designed for PubMed. This powerful tool is capable of tagging various entities within PubMed titles and abstracts, including genes (which include miRNAs), diseases, species, chemicals, cell lines, and mutations. The results generated by PubTator are saved in PubTator format, which can be accessed and read using Python for further analysis and exploration.

Tagging miRNA: The miRNA tagging process involved tokenizing the titles and abstracts while preserving hyphenated words and then vectorized them. Regex was utilized to detect miRNA from the tokens, taking advantage of the well-defined and closely followed nomenclature of miRNA. This nomenclature typically consists of a prefix, such as “miR” (or “miRNA”, “micro-RNA”, “microRNA”) followed by a unique identifying number, which is assigned based on sequence order (e.g., “miR-1”), with exceptions let-7 and lin-4 retaining their names for historical reasons. Additionally, the name might include one suffix such as “-a”, “-1”, “-3p”, or “-5p”. It may also include a prefix that denotes the species, following regular expression was employed to identify miRNAs from retrieved publication titles and abstracts tokens in different formats, and both lower and upper case were considered: [Mm][Ii][Cc]?[Rr][Oo]?[Rr]?[Nn]?[Aa]?-?\d+[a-zA-Z]?-?[12345]?-?[35]?[Pp]?|[Ll][Ee][Tt]-?\d+[a-zA-Z]?-?[35]?[Pp]?, which was supported by a pervious study (14), and were further validated by PubTator gene term labeling. Extracted miRNA from different literature may represent the same miRNA, due to differences in naming conventions, e.g., miR-155, microRNA-155, miR155, miR-155-5p, and others. To ensure accuracy in downstream analysis, miRNAs in different formats were converted into a standardized format, disregarding any extensions such as species prefix, -3p/-5p or genomic suffixes. This approach allows the focus to be on the core miRNA name. Furthermore, to avoid introducing bias, each miRNA was only counted once within an any given literature title/abstract.

### Train-test data set splitting and machine learning analysis

To address the issue of imbalanced data, an equal number of the diabetes studies were paired with the non-diabetes studies for model prediction. Four machine learning models were employed, namely Naïve Bayes (NB), Decision Tree (DT), Random Forest (RF), and Support Vector Machines (SVM). The performance of these models was evaluated using a receiver operating characteristic (ROC) curve and the area under the ROC curve (AUC). To evaluate the models against ground scientific truth, holdout validation was used for model validation.

### Pathway signaling

To explore a comprehensive set of functional annotations of the hub genes, Kyoto Encyclopedia of Genes and Genomes (KEGG) signaling pathway analysis was performed with the gene list using NetworkAnalyst 3.0 (https://www.networkanalyst.ca/). A *P*-value of < 0.05 was considered significant.

## Results

### MiRNAs discovery history in diabetes

**Figure 2:**
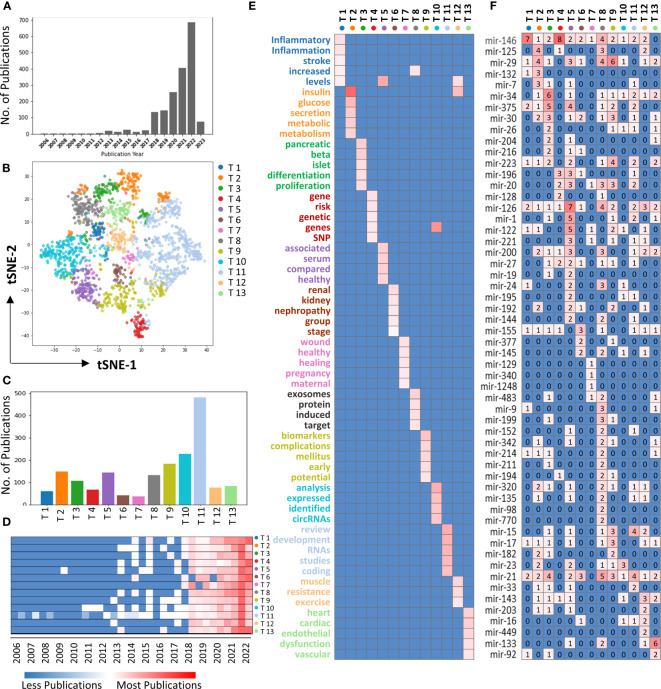
Topic modeling of miRNAs studies in diabetes. **(A)** Bar graph showing the miRNA studies in diabetes conducted each year. **(B)** tSNE plot showing the distribution of 13 topics of miRNAs studies in diabetes. Each dot represents an individual study, and each color corresponds to the indicated topic. **(C)** The bar graph showing the number of publications for each topic. **(D)** Heatmap showing the distributions for each topic over the years. **(E)** Heatmap showing the key topic words for each topic. **(F)** Heatmap showing the miRNAs studies associated with each topic.

A total of 1,818 miRNA studies in the context of diabetes were retrieved from PubMed, spanning the period from January 1, 1993, to March 5, 2023 (**method**). The investigation of miRNA in diabetes began in 2006, with the initial focus being on two specific miRNAs, miR-342, miR-191, and miR-510 in 2009 (15). As shown in **Figure 2A**, research into miRNAs in diabetes has significantly increased since 2018 and continues to grow each year.

### Topic modeling in miRNA studies of diabetes

To investigate the frequently discussed themes in miRNA studies in diabetes research, we performed topic modeling analysis. After applying the inclusion criteria (method), 1,798 studies were selected for topic modeling. A total of 13 distinct topics were identified from the miRNA studies in diabetes research (**Figure 2B**). Topic 11 emerged as the most significant topic among 13 identified topics, followed by Topic 10, while Topic 7 has the least publications (**Figure 2C**). Furthermore, the heatmap indicates that all the topics showed a great increase post 2018 (**Figure 2D**).

The top 5 keywords with the highest weights in each of the 13 topics were shown in **Figure 2E**. Topic 1 was associated with inflammation and stroke, Topic 2 with metabolism, Topic 3 with pancreatic islet beta cells, Topic 4 with gene mutation (such as SNP), Topic 5 with detecting miRNA in serum, Topic 6 with diabetic kidney disease, Topic 7 with wound healing, Topic 8 with exosomes, Topic 9 with identifying miRNA as a biomarker, Topic 10 with relationship with other noncoding RNAs (such as circRNAs), Topic 11 with development, Topic 12 with insulin resistance, and Topic 13 with diabetic heart disease. Furthermore, miRNAs were identified for each of topic (**Figure 2F**), and it was observed that miRNAs trended to cluster together based on the each of the topic, such as miR-146 was studied frequently in Topic 1 and Topic 4, while miR-126 was frequently associated with Topic 5.

### Frequency-distribution of miRNAs in diabetes

A subset of 552 articles with titles containing individual miRNAs was used for machine learning classification. In addition, an equal number of miRNA studies from non-diabetes research were paired as negative controls for the analysis. Analysis of 552 publications of diabetes studies revealed that the top 20 frequently referenced miRNAs are miR-146, miR-21, miR-126, miR-29, miR-375, miR-200, miR-223, miR-34, miR-20, miR-30, miR-122, miR-15, miR-143, miR-155, miR-1, miR-125, miR-133, miR-17, miR-23, and miR-27 (**Supplementary Figure 1A**). Similarly, the top 20 miRNAs from negative control literatures were identified and presented in **Supplementary Figure 1B**.

To gain insight into the overall topic discussed in miRNA studies in both diabetes and non-diabetes research, word clouds were generated using the tokens from 1,798 abstracts. As expected, the most frequently occurring words included “miRNA”, “expression”, “diabetes”, “patient”, and “type”. In contrast, in the non-diabetes literatures, the most frequently used words were “miRNA”, “expression”, and “patient”, which represent general concept related to miRNAs but not associated with any specific diseases (**Supplementary Figures 1C, D**).

### Comparison of different machine learning models predicting diabetes with miRNAs

**Figure 3:**
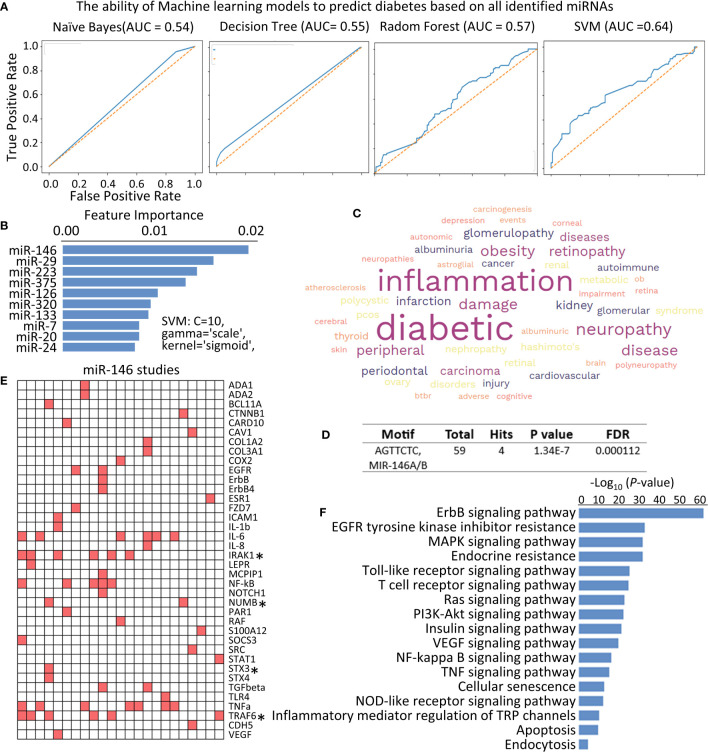
Identifying the function of miR-146 in diabetes. **(A)** Receive operating characteristic (ROC) curves were generated to assess the performance of different machine learning models in predicting diabetes based on all the identified miRNAs. **(B)** Bar graph showing the feature importance scores of key miRNAs measured by the SVM in predicting diabetes. **(C)** WordCloud displaying the key diabetic complications associated with miR-146 function. **(D)** Motif Analysis of identified genes, and total 4 hits are the targets of miR-146 in the context of diabetes studies. **(E)** Heatmap illustrating the presence or absence of key genes in individual studies investigating the role of miR-146 in diabetes. Each column represents an individual study, while each row corresponds to a specific gene mentioned in the study abstracts. The presence of a gene is depicted by red, while the absence is represented by white. Asterisks (*) indicate the genes that are direct targets of miR-146 in the context of diabetes. **(F)** Bar plot showing the KEGG signal pathways influenced by miR-146 in the context of diabetes studies, determined through text mining. The X-axis represents the -log10 (P-value), indicating the significance of pathway enrichment.

To evaluate the predictive potential of miRNAs for diabetes, we utilized four machine learning models (NB, DT, RF, and SVM) as our methodology. These models were employed to predict diabetes based on the complete set of identified miRNAs. The dataset was split into training and testing datasets using holdout splitting methodology. The performance of each model was evaluated, and the results are presented in **Table 1** and **Figure 3A**. Notably, the SVM model, specifically with hyperparameters C = 10, gamma = “scale”, kernel = “sigmoid”, outperformed other models, achieving an accuracy score of 0.606 and an AUC of 0.64.

**Table 1:** Performance comparison of different machine learning models for classifying diabetes with miRNAs.

Model	Predictor	Accuracy	Recall	Precision	MCC	F-score
NB	miRNAs	0.552	0.7368	0.1296	0.1523	0.2205
DT	miRNAs	0.5385	0.5152	0.9444	0.1553	0.6667
RF	miRNAs	0.5656	0.5638	0.4907	0.1293	0.5248
SVM	miRNAs	0.6063	0.5789	0.7130	0.2220	0.6390

Furthermore, we conducted feature importance analysis using the SVM model to identify the most important miRNAs in predicting diabetes. **Figure 3B** shows the key miRNAs that exert the greatest impact on the accuracy of diabetes prediction. Notably, miR-146, along with miR-29, miR-223, miR-375, miR-126, miR-320, miR-133, miR-17, miR-20, and miR-24, play a crucial role in accurately predicting diabetes. Among these miRNAs, miR-146 stands out as the most prominent, as indicated by its importance score of 0.0181, followed by miR-29 with an importance score of 0.01471 (**Figure 3B**). This observation indicates that miR-146 influences the overall performance and accuracy of the SVM model in predicting diabetes and highlights the potential significance of delving deeper into the detailed exploration of miR-146.

### Investigating the Role of miR-146 in diabetes

The high importance score of miR-146 suggests that it plays a pivotal role in distinguishing between individuals with diabetes and those without the condition. A total of 34 diabetes studies focusing on miR-146 were identified and analyzed. A word cloud generated from these studies revealed that miR-146 is potentially involved in various diabetic complications, particularly inflammation and neuropathy (**Figure 3C**), which was consistent with the result that observed in topic modeling (**Figures 2E, F**).

The analysis of individual studies on miR-146 in the context of diabetes yielded a heatmap that showcased the prevalence of several genes, namely *IRAK1*, *TRAF6*, *IL-6*, *TNF-α*, *NUMB*, *EGFR*, and *TGFβ-1*, among others. To further investigate to direct target genes of miR-146, an integrating analysis with TargetScanHuman data set (16) was performed. The results revealed that *IRAK1*, *TRAF6, NUMB*, and *STX3* are direct targets of miR-146 in the context of diabetes (**Figures 3D, E**). In addition, KEGG signal pathways were constructed using the identified gene list obtained from relevant diabetes literature. The resulting analysis highlighted several significant pathways, including the ErbB signaling pathway, Toll-like receptor signaling pathway, NF-kB signaling pathway, Insulin signaling pathway, and TNF signaling pathway (**Figure 3F**). These findings provide valuable insights into the potential mechanisms underlying the involvement of miR-146 in diabetes. The prevalence of specific genes and their direct targeting by miR-146, as well as the identification of relevant signaling pathways, contribute to a more comprehensive understanding of the role of miR-146 in the pathogenesis and progression of diabetes.

## Discussion

In recent years, miRNAs have gained attention as potential biomarkers and therapeutic targets for various diseases, including diabetes (17–19). miRNAs play a crucial role in the regulation of multiple pathways implicated in diabetes pathogenesis, such as insulin secretion, insulin signaling, beta cell function, and glucose homeostasis. Dysregulation of miRNAs has been associated with the development of both type 1 and type 2 diabetes. The current study aimed to summarize our current understanding of miRNAs and their involvement in the development and progression of diabetes and its complications.

We identified 13 crucial areas that highlight the extensive implementation of miRNAs in various domains of diabetes investigation. Notably, miRNA studies encompassed important aspects such as diabetes biomarker research and the exploration of diabetes-related complications, including diabetic inflammation, diabetic cardiovascular diseases, and diabetes kidney disease. One intriguing finding was the identification of miRNA-specific patterns within different domains of diabetes research. For instance, we observed that miR-34 garnered substantial attention in Topic 3, specifically relating to its impact on pancreatic islets/beta cells. This observation aligns with recent review studies, such as the one by Pasquale Mone et al. (20), which emphasized the significant role of miR-34 in regulating pancreatic islets/beta cell function in the context of diabetes. These findings shed light on the multifaceted involvement of miRNAs in diabetes research and emphasize their potential as valuable tools for understanding the pathogenesis and complications of diabetes. By uncovering miRNA-specific patterns in different domains, we provide researchers with valuable insights for further investigations and potential therapeutic interventions.

To unravel potential biomarkers, state-of-art machine learning classifiers were employed. Among the classifiers utilized, the SVM model exhibited exceptional performance, achieving an impressive accuracy score of 0.60. Notably, miR-146 emerged as the most important feature contributing significantly to the accuracy and effectiveness of the prediction model. These findings underscore the potential significance of miR-146 as a key biomarker in the intricate landscape of diabetes.

MiR-146 has been well-documented in human disease (21). And notably, it has emerged as a critical miRNA whose deregulation has been implicated in pathogenesis of diabetes. Most recent review study summarized the implication of miR-146 in type 1 diabetes and type 2 diabetes (22–24). Its significance is underscored by numerous studies that have examined the express levels of miR-146 across various sample types, including whole blood, serum, PBMC, plasma (25–31). These studies collectively demonstrate the involvement of miR-146 in diabetes and highlight its potential as a biomarker for disease detection and monitoring. In our finding, we observed that miR-146 is involved in ErbB signaling, EGFR tyrosine kinase inhibitor resistance, MAPK signaling pathway, Endocrine resistance, Toll like receptor signaling, TNF signaling, NF-kB signaling pathway, etc., consistent with the published studies (22, 32–35).

In addition to miR-146, our study identified the involvement of several other miRNAs that exhibited varying degrees of contribution to the SVM-based diabetes prediction, including miR-29, miR-223, miR-375, miR-126, miR-320, and miR-133, among others. This indicates that these miRNAs play a critical role in diabetes as well. For example, it has been shown that miR-29 is associated with topics 2, 5, 8, and 9, miR-223 is studied in relation to topic 9, and miR-375 demonstrates relevance to topics 3 and 5 (**Figure 2**). These findings are consistent with and supported by previous review studies in the field (36–42).

The utilization of miRNAs as biomarkers and therapeutic targets has the potential to improve the management of diabetes and its associated complications. However, it is worth noting that while the SVM model demonstrated a notable accuracy score of 0.60, there is room for improvement by incorporating additional features such as genes and SNPs. These additional factors can provide a more comprehensive and nuanced understanding of the intricate mechanisms underlying miRNA’s role in diabetes. Considering the multifaceted nature of diabetes, a comprehensive understanding of miRNA’s role, coupled with the integration of additional features, can pave the way for personalized interventions and targeted therapies.

In conclusion, our study revealed a comprehensive understanding of the diverse areas of focus within miRNA research in the context of diabetes. Utilizing SVM with only miRNAs as inputs, we achieved promising results in diabetes prediction, particularly in identifying key miRNAs such as miR-146 as significant players in the context of diabetes. However, it is important to note that further confirmation through additional clinical investigations is necessary to validate and reinforce the findings of this study.

## Data availability statement

The original contributions presented in the study are included in the article/**Supplementary Material**. Further inquiries can be directed to the corresponding author.

## Author contributions

Author Contributions: All authors contributed to the study’s design. RT and JW was in charge of the ML algorithms and performed all statistical analysis and manuscript writing. XL and AD took part in the modeling, and the analytic process had full access to the source data and edited the text. JW, XL and RT created the concept, designed the study, and revised the paper. JW and XL were in charge of the final edit after modifying each aspect of the job. All the authors have read and approved the final version of the paper.

## Supplementary material

Supplementary Figure 1 miRNAs studies in diabetes literatures and non-diabetes literatures **(A, B)** Bar graphs show the top 20 miRNAs studies in diabetes literatures **(A)** and non-diabetes literatures **(B)** from year 2006 to 2023. **(C, D)** Word clouds displaying the frequently used words from abstracts of miRNA studies in diabetes **(C)**, and of miRNA studies in non-diabetes **(D)**.

## References

[1] Guthrie RA Guthrie DW. Pathophysiology of diabetes mellitus. Crit Care Nurs Q (2004) 27(2):113–25. doi: 10.1097/00002727-200404000-00003 15137354

[2] Sen S Chakraborty R. Treatment and diagnosis of diabetes mellitus and its complication: advanced approaches. Mini Rev Med Chem (2015) 15(14):1132–3. doi: 10.2174/138955751514151006154616 26459815

[3] Cloete L. Diabetes mellitus: an overview of the types, symptoms, complications and management. Nurs Stand (2022) 37(1):61–6. doi: 10.7748/ns.2021.e11709 34708622

[4] Wang J You W Jing Z Wang R Fu Z Wang Y. Increased risk of vertebral fracture in patients with diabetes: a meta-analysis of cohort studies. Int Orthop (2016) 40(6):1299–307. doi: 10.1007/s00264-016-3146-y 27029481

[5] Perron MP Provost P. Protein interactions and complexes in human microRNA biogenesis and function. Front Biosci (2008) 13:2537–47. doi: 10.2741/2865 PMC2901379 17981733

[6] Macfarlane LA Murphy PR. MicroRNA: biogenesis, function and role in cancer. Curr Genomics (2010) 11(7):537–61. doi: 10.2174/138920210793175895 PMC3048316 21532838

[7] Ardekani AM Naeini MM. The role of microRNAs in human diseases. Avicenna J Med Biotechnol (2010) 2(4):161–79. PMC3558168 23407304

[8] Dwivedi S Purohit P Sharma P. MicroRNAs and diseases: promising biomarkers for diagnosis and therapeutics. Indian J Clin Biochem (2019) 34(3):243–5. doi: 10.1007/s12291-019-00844-x PMC6660526 31391712

[9] Fernandez-Valverde SL Taft RJ Mattick JS. MicroRNAs in beta-cell biology, insulin resistance, diabetes and its complications. Diabetes (2011) 60(7):1825–31. doi: 10.2337/db11-0171 PMC3121441 21709277

[10] LaPierre MP Stoffel M. MicroRNAs as stress regulators in pancreatic beta cells and diabetes. Mol Metab (2017) 6(9):1010–23. doi: 10.1016/j.molmet.2017.06.020 PMC5605735 28951825

[11] Grieco GE Brusco N Licata G Fignani D Formichi C Nigi L. The Landscape of microRNAs in betaCell: Between Phenotype Maintenance and Protection. Int J Mol Sci (2021) 22(2). doi: 10.3390/ijms22020803 PMC7830142 33466949

[12] Isaacs SR Wang J Kim KW Yin C Zhou L Mi QS. MicroRNAs in type 1 diabetes: complex interregulation of the immune system, beta cell function and viral infections. Curr Diabetes Rep (2016) 16(12):133. doi: 10.1007/s11892-016-0819-2 27844276

[13] Wei CH Allot A Leaman R Lu Z. PubTator central: automated concept annotation for biomedical full text articles. Nucleic Acids Res (2019) 47(W1):W587–W93. doi: 10.1093/nar/gkz389 PMC6602571 31114887

[14] Friedrich J Hammes HP Krenning G. miRetrieve-an R package and web application for miRNA text mining. NAR Genom Bioinform (2021) 3(4):lqab117. doi: 10.1093/nargab/lqab117 34988440 PMC8696973

[15] Hezova R Slaby O Faltejskova P Mikulkova Z Buresova I Raja KR. microRNA-342, microRNA-191 and microRNA-510 are differentially expressed in T regulatory cells of type 1 diabetic patients. Cell Immunol (2010) 260(2):70–4. doi: 10.1016/j.cellimm.2009.10.012 19954774

[16] McGeary SE Lin KS Shi CY Pham TM Bisaria N Kelley GM. The biochemical basis of microRNA targeting efficacy. Science (2019) 366(6472). doi: 10.1126/science.aav1741 PMC7051167 31806698

[17] Jin ZQ. MicroRNA targets and biomarker validation for diabetes-associated cardiac fibrosis. Pharmacol Res (2021) 174:105941. doi: 10.1016/j.phrs.2021.105941 34656765

[18] He X Kuang G Wu Y Ou C. Emerging roles of exosomal miRNAs in diabetes mellitus. Clin Transl Med (2021) 11(6):e468. doi: 10.1002/ctm2.468 34185424 PMC8236118

[19] Vasu S Kumano K Darden CM Rahman I Lawrence MC Naziruddin B. MicroRNA signatures as future biomarkers for diagnosis of diabetes states. Cells (2019) 8(12). doi: 10.3390/cells8121533 PMC6953078 31795194

[20] Mone P de Donato A Varzideh F Kansakar U Jankauskas SS Pansini A. Functional role of miR-34a in diabetes and frailty. Front Aging (2022) 3:949924. doi: 10.3389/fragi.2022.949924 35923683 PMC9340262

[21] Li L Chen XP Li YJ. MicroRNA-146a and human disease. Scand J Immunol (2010) 71(4):227–31. doi: 10.1111/j.1365-3083.2010.02383.x 20384865

[22] Alipoor B Ghaedi H Meshkani R Torkamandi S Saffari S Iranpour M. Association of miR-146a expression and type 2 diabetes mellitus: A meta-analysis. Int J Mol Cell Med (2017) 6(3):156–63. doi: 10.22088/acadpub.BUMS.6.3.156 PMC5898639 29682487

[23] Ghaffari M Razi S Zalpoor H Nabi-Afjadi M Mohebichamkhorami F Zali H. Association of microRNA-146a with type 1 and 2 diabetes and their related complications. J Diabetes Res (2023) 2023:2587104. doi: 10.1155/2023/2587104 36911496 PMC10005876

[24] Liu XS Fan B Szalad A Jia L Wang L Wang X. MicroRNA-146a mimics reduce the peripheral neuropathy in type 2 diabetic mice. Diabetes (2017) 66(12):3111–21. doi: 10.2337/db16-1182 PMC5697943 28899883

[25] Huang YN Chiang SL Lin YJ Liu SC Li YH Liao YC. Long, noncoding RNA SRA induces apoptosis of beta-cells by promoting the IRAK1/LDHA/lactate pathway. Int J Mol Sci (2021) 22(4). doi: 10.3390/ijms22041720 PMC7914996 33572095

[26] Liu Y Ma M Yu J Ping F Zhang H Li W. Decreased Serum microRNA-21, microRNA-25, microRNA-146a, and microRNA-181a in Autoimmune Diabetes: Potential Biomarkers for Diagnosis and Possible Involvement in Pathogenesis. Int J Endocrinol (2019) 2019:8406438. doi: 10.1155/2019/8406438 31582977 PMC6754900

[27] Assmann TS Duarte GCK Brondani LA de Freitas PHO Martins EM Canani LH. Polymorphisms in genes encoding miR-155 and miR-146a are associated with protection to type 1 diabetes mellitus. Acta Diabetol (2017) 54(5):433–41. doi: 10.1007/s00592-016-0961-y 28101643

[28] Wang G Gu Y Xu N Zhang M Yang T. Decreased expression of miR-150, miR146a and miR424 in type 1 diabetic patients: Association with ongoing islet autoimmunity. Biochem Biophys Res Commun (2018) 498(3):382–7. doi: 10.1016/j.bbrc.2017.06.196 28733034

[29] Duan X Zhan Q Song B Zeng S Zhou J Long Y. Detection of platelet microRNA expression in patients with diabetes mellitus with or without ischemic stroke. J Diabetes Complications (2014) 28(5):705–10. doi: 10.1016/j.jdiacomp.2014.04.012 24908639

[30] Baldeon RL Weigelt K de Wit H Ozcan B van Oudenaren A Sempertegui F. Decreased serum level of miR-146a as sign of chronic inflammation in type 2 diabetic patients. PloS One (2014) 9(12):e115209. doi: 10.1371/journal.pone.0115209 25500583 PMC4264887

[31] Balasubramanyam M Aravind S Gokulakrishnan K Prabu P Sathishkumar C Ranjani H. Impaired miR-146a expression links subclinical inflammation and insulin resistance in Type 2 diabetes. Mol Cell Biochem (2011) 351(1-2):197–205. doi: 10.1007/s11010-011-0727-3 21249428

[32] Lee HW Khan SQ Khaliqdina S Altintas MM Grahammer F Zhao JL. Absence of miR-146a in Podocytes Increases Risk of Diabetic Glomerulopathy *via* Up-regulation of ErbB4 and Notch-1. J Biol Chem (2017) 292(2):732–47. doi: 10.1074/jbc.M116.753822 PMC5241746 27913625

[33] Rusca N Monticelli S. MiR-146a in immunity and disease. Mol Biol Int (2011) 2011:437301. doi: 10.4061/2011/437301 22091404 PMC3200075

[34] Peng X He F Mao Y Lin Y Fang J Chen Y. miR-146a promotes M2 macrophage polarization and accelerates diabetic wound healing by inhibiting the TLR4/NF-kappaB axis. J Mol Endocrinol (2022) 69(2):315–27. doi: 10.1530/JME-21-0019 35604113

[35] Barutta F Bellini S Mastrocola R Bruno G Gruden G. MicroRNA and microvascular complications of diabetes. Int J Endocrinol (2018) 2018:6890501. doi: 10.1155/2018/6890501 29707000 PMC5863305

[36] Dalgaard LT Sorensen AE Hardikar AA Joglekar MV. The microRNA-29 family: role in metabolism and metabolic disease. Am J Physiol Cell Physiol (2022) 323(2):C367–C77. doi: 10.1152/ajpcell.00051.2022 35704699

[37] Hourigan ST Solly EL Nankivell VA Ridiandries A Weimann BM Henriquez R. The regulation of miRNAs by reconstituted high-density lipoproteins in diabetes-impaired angiogenesis. Sci Rep (2018) 8(1):13596. doi: 10.1038/s41598-018-32016-x 30206364 PMC6133943

[38] Li X. MiR-375, a microRNA related to diabetes. Gene (2014) 533(1):1–4. doi: 10.1016/j.gene.2013.09.105 24120394

[39] Ma Y Liu H Wang Y Xuan J Gao X Ding H. Roles of physical exercise-induced MiR-126 in cardiovascular health of type 2 diabetes. Diabetol Metab Syndr (2022) 14(1):169. doi: 10.1186/s13098-022-00942-6 36376958 PMC9661802

[40] Nigi L Grieco GE Ventriglia G Brusco N Mancarella F Formichi C. MicroRNAs as regulators of insulin signaling: research updates and potential therapeutic perspectives in type 2 diabetes. Int J Mol Sci (2018) 19(12). doi: 10.3390/ijms19123705 PMC6321520 30469501

[41] Kim M Zhang X. The profiling and role of miRNAs in diabetes mellitus. J Diabetes Clin Res (2019) 1(1):5–23. doi: 10.33696/diabetes.1.003 32432227 PMC7236805

[42] Feng J Xing W Xie L. Regulatory roles of microRNAs in diabetes. Int J Mol Sci (2016) 17(10). doi: 10.3390/ijms17101729 PMC5085760 27763497

